# The Mongrel College of the Future

**Published:** 1887-06

**Authors:** 


### THE MONGREL COLLEGE OF THE FUTURE.

In the "*Hahnemannian*" *Monthly* for April, 1887, page 239, we find this announcement as to what a mongrel medical college should be! It is neat: "Briefly, then, the homœopathic physician should be taught at college what Homœopathy cannot do as carefully as he is instructed in what it can do. He should also be taught what allopathy can do, and just as thoroughly instructed as to what it cannot do. But especially should he be instructed with the utmost pains in those facts and principles which alone can be his guide and his justification when he is called upon to lay down his homœopathic medicine case and call in the aid of other measures, so that he may do it fearlessly in the sight of men and angels."

In contradiction to the assertion here made that allopathy can do something which Homœopathy cannot do, we find on the previous page (238) of this same journal this declaration: "The homœopathist knows that the so-called allopathic cures are not cures at all in the sense in which he understands that term."

